# Insulin-like Growth Factor Binding Protein-2 (IGFBP2) Is a Key Molecule in the MACC1-Mediated Platelet Communication and Metastasis of Colorectal Cancer Cells

**DOI:** 10.3390/ijms222212195

**Published:** 2021-11-11

**Authors:** Reza Haschemi, Dennis Kobelt, Elisabeth Steinwarz, Martin Schlesinger, Ulrike Stein, Gerd Bendas

**Affiliations:** 1 Pharmaceutical Department, University of Bonn, An der Immenburg 4, 53121 Bonn, Germany; rhas@uni-bonn.de (R.H.); elisabeth.steinwarz@gmail.com (E.S.); martin.schlesinger@uni-bonn.de (M.S.); 2 Experimental and Clinical Research Center, Charité-Universitätsmedizin Berlin and Max-Delbrück-Center for Molecular Medicine, Robert-Rössle-Straße 10, 13125 Berlin, Germany; Dennis.Kobelt@epo-berlin.com; 3 German Cancer Consortium, Im Neuenheimer Feld 280, 69120 Heidelberg, Germany

**Keywords:** colorectal cancer, IGFBP2, MACC1, metastasis, platelets

## Abstract

Tumor cell crosstalk with platelets and, subsequently, their activation are key steps in hematogenous tumor metastasis. MACC1 is an oncogene involved in molecular pathogenesis of colorectal cancer (CRC) and other solid tumor entities, mediating motility and metastasis, making MACC1 an accepted prognostic biomarker. However, the impact of MACC1 on platelet activation has not yet been addressed. Here, we investigated the activation of platelets by human CRC cells upon MACC1 modulation, indicated by platelet aggregation and granule release. These approaches led to the identification of insulin-like growth factor binding protein-2 (IGFBP2) as a functional downstream molecule of MACC1, affecting communication with platelets. This was confirmed by an shRNA-mediated IGFBP2 knockdown, while maintaining MACC1 activity. Although IGFBP2 displayed an attenuated platelet activation potential, obviously by scavenging IGF-I as a platelet costimulatory mediator, the MACC1/IGFBP2 axis did not affect the thrombin formation potential of the cells. Furthermore, the IGFBP2/MACC1-driven cell migration and invasiveness was further accelerated by platelets. The key role of IGFBP2 for the metastatic spread in vivo was confirmed in a xenograft mouse model. Data provide evidence for IGFBP2 as a downstream functional component of MACC1-driven metastasis, linking these two accepted oncogenic biomarkers for the first time in a platelet context.

## 1. Introduction

Metastasis-associated in colon cancer (MACC) 1 has been identified as a key player in CRC malignancy. It is meanwhile accepted as a prognostic and predictive biomarker for tumor progression and metastasis in a great variety of more than 20 solid cancers [1,2]. MACC1 possesses manifold activities, either as a transcription factor or as a cellular adaptor protein, in driving increased cell proliferation and growth. It also contributes to therapy resistance or to dysregulation of apoptosis [3,4]. Most attention has been given to its pro-metastatic and invasive activities in solid tumors. MACC1 transcriptionally activates the epithelial–mesenchymal transition (EMT) associated with modifications of the extracellular matrix, resulting in a higher invasiveness of cells [5]. Clinically, this is reflected by an accumulation of MACC1 at the invasive front of tumor cells [6]. This “polarized” activity of MACC1 at the invasion front refers to a probable impact of MACC1 on cell communication with the cellular or noncellular microenvironment. Communication with the tumor microenvironment, e.g., by the secretion of bioactive molecules, is a prerequisite to fulfill the different steps in the complex cascade of hematogenous metastasis. Especially in the blood passage, the tumor cells rely on a tight interaction with cellular components to form a permissive milieu for survival and protection from immune surveillance and shear forces [7,8,9].

Platelets represent the most important blood components that tumors cells encounter when entering the blood system. Within minutes after intravasation, tumor cells contact and activate platelets. The impact of platelets to protect tumor cells in the metastatic process is, meanwhile, an accepted key issue to account for the metastatic success [9,10,11]. The mechanisms of platelets to protect disseminated tumor cells are versatile, including the formation of a protective shield around the tumor cells and avoiding attacks by cells of the immune surveillance [12,13,14], release of growth factors, secretion of chemokines to recruit myeloid cells [15], or to support metastatic niche formation by mediating cell adhesion, extravasation, and angiogenesis [16,17,18,19,20,21,22,23]. Despite the manifold pro-metastatic activities of MACC1, its impact on platelet activation and interaction by the respective tumor cells has not yet been investigated.

In terms of hematogenous metastasis, another oncogene, namely insulin-like growth factor binding protein-2 (IGFBP2), comes into consideration, which has surprisingly neither been associated with the oncogene MACC1, nor with tumor-cell-induced platelet activation. IGFBP2 has been described to be overexpressed by CRC patients, thus IGFBP2 levels were positively related to tumor load and clinical pathological parameters [24]. Meanwhile, IGFBP2 is regarded as a biomarker in multiple solid cancers indicating high tumor proliferation, invasiveness, and metastatic activities [25]. IGFBPs comprise a family of six members, all of them possessing high-affinity binding to insulin-like growth factors IGF-I and IGF-II, and thus the ability to modulate the biological functions and activities of these growth factors [26]. IGFBP2 has an outstanding role among the IGFBPs in oncology; besides the transport and binding function for IGF-I and IGF-II, it has several pro-tumorigenic activities independent of IGF, but obviously related to integrins [27]. Notably, IGFBP2 has been associated with chemoresistance in cancer cells [28,29], e.g., by inducing an EMT program in pancreatic or other cancer cells, respectively [28,30]. Deciphering the molecular mechanisms of IGFBP2 in CRC, its transcriptional activation via NF-κB leads to an upregulation of the Wnt/β-catenin signaling pathway [31], a common feature shared with MACC1 activity. Nevertheless, a link between IGFBP2 and MACC1 has never been investigated to drive malignancy of tumors.

Here, we aim to elucidate a potential impact of MACC1 activity in tumor cells in relation to the platelet activation potential, anticipating MACC1 as a promoting factor for intensified platelet tumor cell interaction, hence enlarging knowledge on the pro-metastatic activity of MACC1 to the level of blood cell interaction. Following platelet activation by MACC1-positive SW620 CRC cells and their MACC1 KO variant, we identified IGFBP2 upregulated in the transcriptome, as well as in the secretome of the supernatant of the MACC1 cells. We reveal IGFBP2 as a downstream component of MACC1, affecting platelet activation potential and increasing cell mobility, invasiveness, and metastatic activity. This study is the first report on an MACC1/IGFPB2 activation axis, providing insights into molecular downstream activities of MACC1, with IGFBP2 emerging as a promising target in MACC1-driven malignancy for therapeutic interference.

## 2. Results

### 2.1. The Impact of MACC1 on Tumor-Cell-Induced Platelet Activation

**Figure 1:**
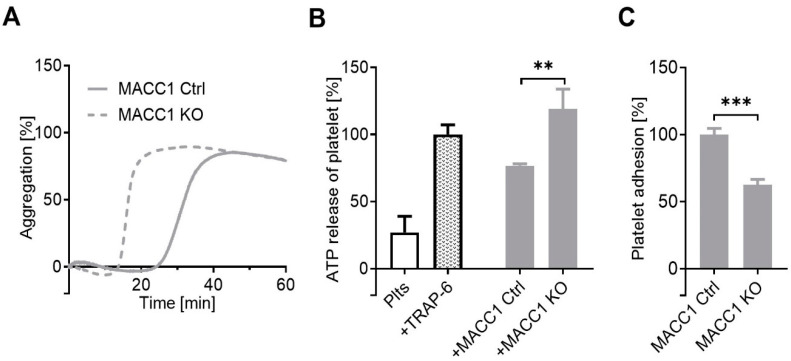
Dependency of platelet activation by SW620 cells from the MACC1 expression. (**A**) Representative curves of platelet aggregation by light transmission measurement after contact with MACC1-positive SW620 Ctrl cells or the MACC1 KO variant. (**B**) ATP release from platelet dense granule induced by SW620 Ctrl and MACC1 KO cells. (**C**) Platelet adhesion to a cell layer of SW620 Ctrl or MACC1 KO cells exclude a diminished cell contact formation as the reason for lower platelet activation by the MACC1-positive cells. ** *p* < 0.01, *** *p* < 0.001.

Although MACC1 possesses various modes of action to foster tumor metastasis, a functional insight whether and how tumor cell communication with the cellular microenvironment is affected by MACC1 remains elusive. This is especially true with respect to contact formation and, thus, activation of platelets. Notably, an impact of MACC1 on tumor cell/platelet communication has not been addressed yet. To focus on this at a functional level, we selected the endogenously MACC1-positive SW620 CRC cell line. Taking an established MACC1 knockout (SW620 MACC1 KO) model, compared to SW620 MACC1 Ctrl (harboring only Cas9) cells [3], we analyzed their interaction with human platelets. Platelet aggregation is considered as a valuable readout to monitor tumor-cell-induced platelet activation. It was surprising to detect an even lower activity of SW620 MACC1 Ctrl cells compared to the MACC1 KO cells to induce platelet aggregation. This was reflected by a decelerated onset of aggregation by the MACC1 Ctrl cells compared to MACC1 KO cells, but finally reaching an identical aggregation level (Figure 1A).

Quantification of platelet ATP dense granule release is another indicator for platelet activation upon tumor cell contact. Untreated platelets were considered as negative and platelets activated by the thrombin receptor activator TRAP-6 as positive controls, respectively. Confirming the findings on platelet aggregation, the MACC1 KO cells caused a significantly higher ATP release from the platelets than the SW620 MACC1 Ctrl cells (Figure 1B), contradicting a clear MACC1/platelet activation axis. However, these findings prompted the search for the underlying molecular mechanism.

### 2.2. A Component of the Supernatant Is Responsible for Attenuated Platelet Activation by SW620 Cells

To search for a molecular mechanism by which the MACC1 KO variant of SW620 cells differs from the MACC1 Ctrl cells in the platelet activation potential, it is conceivable that the physical contact formation between cells and platelets or the appearance of a soluble inhibitory component in the MACC1-positive cells are responsible for the observed differences. To engage the first option, we detected the direct interaction of platelets with the indicated tumor cells by a static microscopic approach, analyzing the attachment of fluorescently labeled platelets onto a confluent cell layer. It became evident that the SW620 MACC1 Ctrl cells have a significantly higher platelet binding capacity compared to the MACC1 KO variant (Figure 1C). Thus, the deficit in platelet contact formation of SW620 cells seems to be an unlikely reason for the attenuated platelet activation.

**Figure 2:**
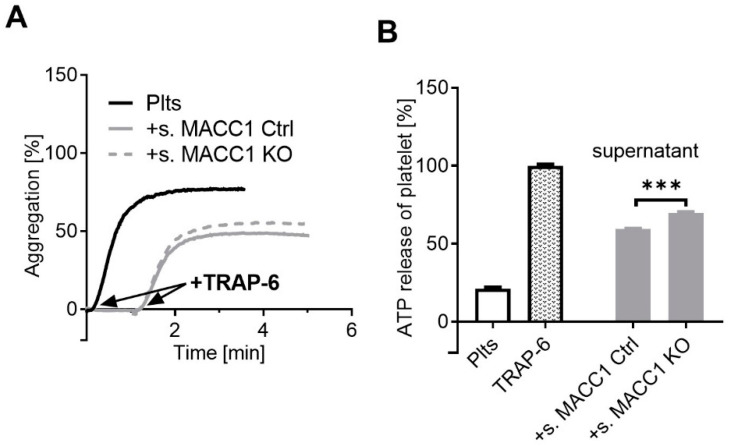
The cell supernatant of SW620 cells interferes with platelet activation. (**A**) Representative traces showing platelet aggregation in response to TRAP-6 (black) and its inhibition by supernatant of SW620 MACC1 Ctrl cells (solid grey line) and a lower inhibition by MACC1 KO cells (dashed grey line), respectively. (**B**) Quantification of ATP release from resting platelets, platelets activated with TRAP-6, and co-incubated with supernatant of SW620 MACC1 Ctrl (s. MACC1 Ctrl) and MACC1 KO (s. MACC1 KO) cells, respectively. Data indicate a soluble inhibitory compound in the cell supernatant that is more potent in the SW620 Ctrl cells. *** *p* < 0.001.

To evaluate whether a soluble component of the SW620 cells interferes with platelet activation, we checked the supernatant of SW620 cells, either of the MACC1 Ctrl or of the MACC1 KO variant, with respect to probable inhibitory properties in platelet activation. First, we selected a low amount of the thrombin receptor agonist TRAP-6, which induces an incomplete platelet aggregation of about 70% of the maximal effect. Subsequently, this amount of TRAP-6 was added to those platelets that had been preincubated with the supernatant of the SW620 cell populations. While each of the supernatants alone had no effect on the platelet activation, they diminished the TRAP-6-induced platelet aggregation to a range of about 50%. Notably, the supernatant of MACC1 Ctrl cells had a slightly higher inhibitory effect than that of the MACC1 KO cells (Figure 2A). An inhibitory effect of the SW620 supernatant on platelet activation became even more clear when analyzing the platelet ATP release (Figure 2B). The effect of TRAP-6 was attenuated by each of the indicated cell supernatants, but MACC1 Ctrl cells were significantly more inhibitory than the MACC1 KO variant. Although the molecular basis for these effects remained open at this point, the findings confirmed a soluble component in the cell supernatant that interferes with platelet activation. This component was secreted in a higher concentration by MACC1-expressing cells.

### 2.3. IGFBP2, Overexpressed by MACC1 Activity, Is Responsible for Attenuated Platelet Activation

To search for molecular candidates that were deregulated in the SW620 cells upon the MACC1 knockout, we started a whole transcriptome analysis of the cells. Upon the most significantly deregulated genes, we selected those components which could have a functional interplay with the platelet activation process. Insulin-like growth factor binding protein-2 (IGFBP2) displayed a 2.86-fold reduction upon knockdown of MACC1 and, thus, appeared as the most evident candidate, which is deregulated in association with MACC1 having a relevance in a platelet context. IGFBP2 is a known factor for malignancy and aggressiveness in different tumor entities regarded as a biomarker, which seems to be in line with the biomarker function of MACC1. Furthermore, as a modulator of IGF-I or IGF-II, it appears likely that IGFBP2 might affect platelet activation, since platelet-derived IGF-I is a known coactivator for platelet aggregation and secretion [32,33,34].

**Figure 3:**
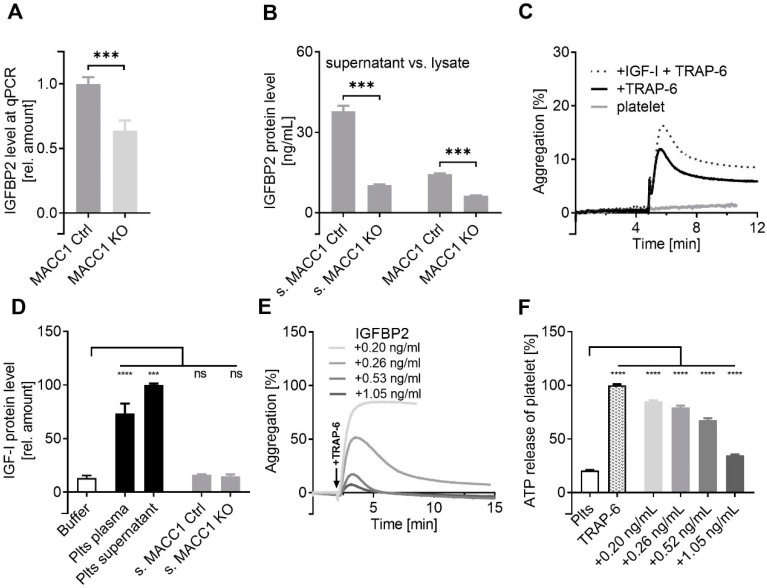
MACC1 activity is directly related to IGFBP2 expression, which interferes with platelet activation. The downregulation of IGFBP2 in the MACC1 KO variant of SW620 cells was indicated at the mRNA level by qPCR (**A**) and confirmed at the protein level by ELISA (**B**). ELISA data confirm the lower expression of IGFBP2 by the MACC1 KO variant of SW620 cells, either in supernatant (s. MACC1 Ctrl vs. s.MACC1 KO, left columns) and at a lower level in the cell lysate (right columns). (**C**) IGF-I is costimulatory for platelet activation and accelerates the effect of 7.5 µM TRAP-6 to induce platelet aggregation. (**D**) IGF-I is associated with platelets and found in platelet plasma or supernatant after activation, but not in considerable amounts in supernatant of SW620 cells. (**E**) Recombinant IGFBP2 is able to block concentration-dependently the activity of TRAP-6 to induce platelet aggregation and (**F**), significantly, the platelet ATP release. *** *p* < 0.001; **** *p* < 0.0001.

To validate the transcriptome findings, we analyzed the IGFBP2 expression at the mRNA level by qPCR (Figure 3A). Notably, IGFBP2 was downregulated at the mRNA level by 40% when MACC1 was missing. This refers to a certain, but not exclusive, transcriptional control of IGFBP2 by MACC1. The IGFBP2 protein followed this trend, as shown by ELISA (Figure 3B). While the IGFBP2 protein level is higher in the cell supernatant compared to lysate, data confirm a significant downregulation of IGFBP2 in the MACC1 KO variant. To check whether a probable relationship between MACC1 and IGFBP2 exists in other CRC cell lines too, we performed a qPCR study considering 10 further CRC cell lines. A positive expression correlation between MACC1 and IGFBP2 was confirmed in LoVo, HCT15, HCT116, SW48, LS174T, WiDr, HCA-7, DLD-1, SW403, and COLO205 cells with a Pearson r = 0.7715, *p* = 0.009, as indicated in Supplementary Figure S1.

The pro-tumorigenic activities of IGFBP2 are either based on IGF-independent (canonical) mechanisms or on modulating the activities of IGF-I and/or IGF-II by serving as a transporter or binding transmitter for them. The latter aspect appears highly relevant for platelet activation, considering IGF-I as a co-stimulator for platelet activation, when reinforcing the effect of TRAP-6 (Figure 3C). As indicated, IGF-I was detectable in significant amounts in both platelet plasma and platelet supernatant, but did not originate from the indicated tumor cells (Figure 3D). However, there are neither evident data available on IGFBP2 in relation to platelets, nor for IGFBP2 acting as a soluble antagonist for IGF-I.

To evaluate whether IGFBP2 can antagonize platelet activation, we again performed platelet aggregation by TRAP-6 addition, and used increasing amounts of recombinant IGFBP2 instead of the cell supernatants. Platelet pre-activation was dose-dependently attenuated by IGFBP2, with a maximal reduction at a concentration of about 1 ng IGFBP2/mL (Figure 3E). Confirming these findings, platelet ATP release assay displayed significantly attenuated ATP release by the addition of IGFBP2 at different concentrations (Figure 3F).

**Figure 4:**
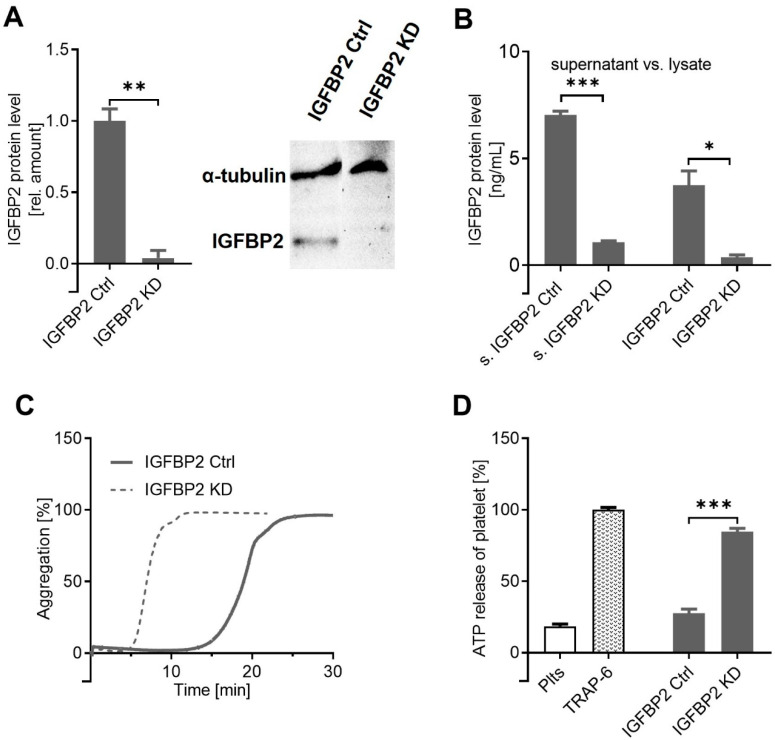
IGFBP2 controls platelet activation by SW620 cells. (**A**) The shRNA-mediated knockdown of IGFBP2 in SW620 cells was confirmed by Western blot, compared to nontargeted knockdown IGFBP2 Ctrl, and quantified by IGFBP2 ELISA using cell supernatants (left columns) and cell lysates (right columns) (**B**). (**C**) The knockdown of IGFBP2 in (MACC1-positive) SW620 cells restored the activation potential to platelets in the aggregation assay, or in ATP release (**D**). * *p* < 0.05; ** *p* < 0.01; *** *p* < 0.001.

To decouple MACC1 and IGFBP2 activities, we performed an IFGBP2 knockdown approach in the SW620 cells (Figure 4A) in an intact MACC1 background. Significant IGFBP2 knockdown was confirmed by ELISA, analyzing the cell supernatant and the cell lysates (Figure 4B). Remarkably, the IGFBP2 KD variant of SW620 exhibited a high platelet activation potential, indicated by a faster induction of aggregation (Figure 4C) and significantly higher amounts of ATP released from platelet granules compared to the SW620 control (Figure 4D). Notably, this confirms the above findings on IGFBP2 on platelet activation by SW620 cells; it more importantly revealed IGFBP2 as a functional downstream component of MACC1 in these cells. To consider these finding in a broader context, the general question arose of whether and how IGFBP2 acts as a downstream component of MACC1 activity and how these two known oncogenic markers are in a functional relationship.

### 2.4. IGFBP2 Is a Functional Downstream Component of MACC1 with Impact on Cell Dynamics

**Figure 5:**
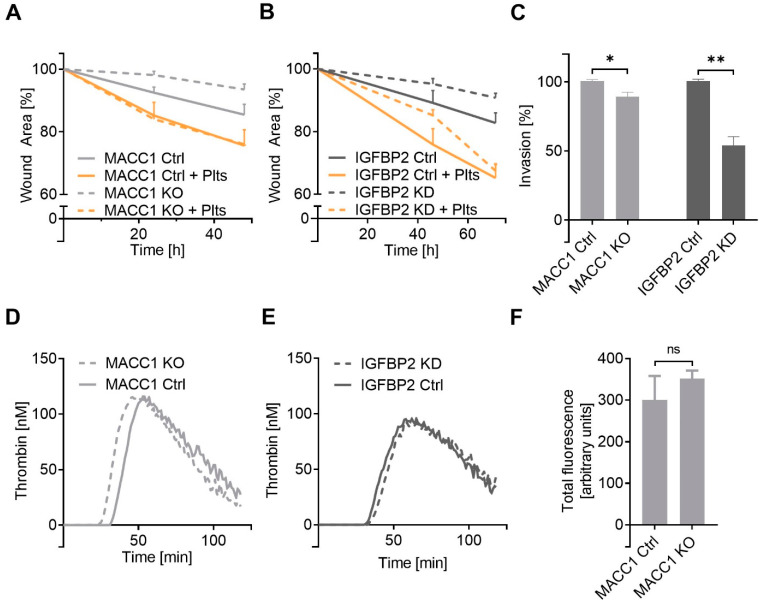
The impact of IGFBP2 as a functional downstream component of MACC1 on SW620 cell dynamics. (**A,B**) Detection of cell migratory dynamics in a 2D wound healing assay comparing the impact of MACC1 (**A**) and IGFBP2 (**B**) on migration and the effect of platelets to accelerate dynamic properties. (**C**) Analyzing cell invasion in a transmigration assay and the impact of MACC1 knockout or IGFBP2 knockdown. (**D,E**) Analyzing the impact of MACC1 KO or IGFBP2 KD on cell capability to induce coagulation indicates no differences in thrombin formation upon MACC1 KO (**D**) or IGFBP2 KD (**E**) when compared to the respective control cells. (**F**) Flow cytometry data confirm that the knockout of MACC1 in SW620 cells has no impact on tissue factor expression. * *p* < 0.05; ** *p* < 0.01, ns = non-significant.

To focus on the MACC1-related IGFBP2 activities on cell characteristics, next, we investigated cell dynamics by following cell migration in a 2D wound healing assay and in a cell invasion approach. The knockout of MACC1 in SW620 cells is associated with a mitigated cell migratory dynamic (Figure 5A), confirming previous findings on the increased cell motility of MACC1-positive cells. Remarkably, the knockdown of IGFBP2 in the MACC1-positive cells led to an identical appearance (Figure 5B), indicating IGFBP2 as a functional downstream component of MACC1 mediating cell migration. Furthermore, the preincubation of these cells with platelets strongly increased the 2D migratory properties of all cells. Notably, the resulting higher level of cell migration induced by the platelets was identical, comparing the MACC1 KO and MACC1 Ctrl cells, or the IGFBP2 KD variant with the control cells, respectively (Figure 5A,B). Despite the differences in platelet activation shown above, MACC1 KO and IGFBP2 KD cells obtained a higher motility “push” by platelets. 

This was also supported by the transmigration data (Figure 5C), indicating that the higher invasiveness of MACC1 cells is associated with IGFBP2 activity. The loss of MACC1 was related to a reduced invasion, while the knockdown of IGFBP2 in the presence of MACC1 more evidently reduced invasive properties, emphasizing IGFBP2 as the key molecule for increased cell dynamics.

Next, we took a closer look into the thrombogenic characteristics of these cells to reveal a more general issue affecting metastasis. Platelet activation is a vital part that contributes to tumor-cell-induced coagulation, but thrombin formation by tissue factor/FVII pathway appears as the superior mechanism to foster coagulation, and thus metastasis. Notably, MACC1 KO and the IGFBP2 KD cells displayed a nearly identical thrombin generation capacity compared to their respective control cells (Figure 5D,E). This is supported by flow cytometry data that the knockout of MACC1 has no consequences for the tissue factor expression profile compared to the control cells (Figure 5F). These data confirm that the indicated lower platelet activation by the MACC1/IGFBP2 axis has no functional consequences on coagulability, and thus do not contradict the higher metastatic potential associated with MACC1. To confirm the relevance of the MACC1/IGFBP2 functional axis for metastasis, subsequently, we performed in vivo studies on experimental metastasis in mice.

### 2.5. IGFBP2 Is the Metastatic Factor In Vivo

**Figure 6:**
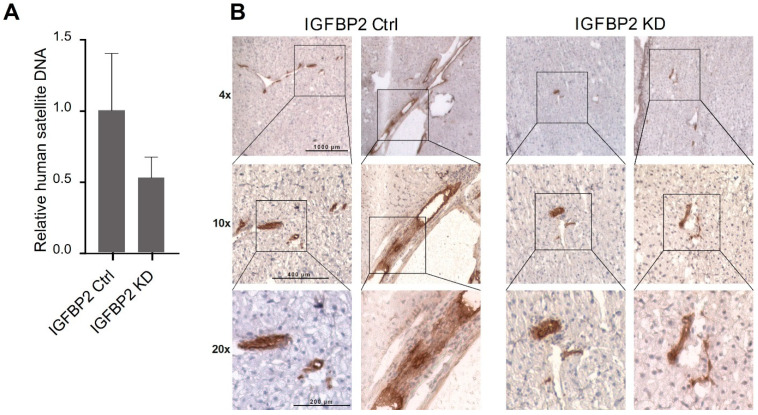
IGFBP2 is the metastatic factor in vivo. After xenotransplantation of SW620 control or SW620 IGFBP2 KD cells, metastasis formation was inhibited by IGFBP2 knockdown, as shown by (**A**) human satellite DNA and (**B**) immunohistochemistry staining of human cytokeratin 19.

To test the effect of IGFBP2 on the ability of cells to form metastasis in vivo, we used the IGFBP2 KD clone of SW620 cells possessing high MACC1 gene expression and compared them with the respective control cells with respect to metastatic behavior. Animals were xenotransplanted with these cells into the spleen and monitored for tumor growth and metastasis formation. Animals were killed when the ethical endpoint was reached by first animals, which corresponded roughly to tumor volumes of 0.5 mm^3^. The livers were collected and subjected to molecular analysis. After cryosectioning and DNA isolation, human satellite DNA load in the murine liver as a marker for the human cell number was quantified. Compared to the control cells, human DNA load was reduced upon IGFBP2 knockdown (Figure 6A). The reduced amount of metastases in the liver was confirmed by immunohistochemistry for human cytokeratin 19 (Figure 6B). The evidently reduced amount of human cells in the mouse liver upon IGFBP2 knockdown clearly indicated the impact of IGFBP2 on reinforcing metastatic behavior of SW620 cells.

## 3. Discussion

In the present study, we provide evidence that IGFBP2 is a functional downstream component of MACC1 in CRC cells, inducing increased cell mobility and metastasis. Thus, MACC1 and IGFBP2, two accepted biomarkers for increased malignancy, invasiveness, and metastasis of various solid tumors, were shown for the first time in a combined approach and in their functional relation in this cellular assay. Although we cannot provide all details on the molecular relationship of MACC1 and IGFBP2, and the IGFBP2 mode of action in this axis, the data represent a new piece of the puzzle in our general understanding of the MACC1 function in malignancy.

IGFBP2 was originally discovered and identified in an oncological context as a regulator of IGF functions in the pericellular space [35]. During the last two decades, IGFBP2 has been reported to be aberrantly expressed in a broad range of cancers and associated with the promotion of several key oncogenic processes. In these terms, the role of IGFBP2 as an oncogene goes far beyond the regulation of IGF activities. It also refers to an IGF-independent mode of action showing an IGFBP2-driven oncogenic network of intracellular and nuclear regulatory activities. The detailed mechanisms underlying those tumorigenic functions still remain to be elucidated [35].

IGFBP2 was strongly expressed and evident in the supernatant of MACC1-driven SW620 cells and obviously displays both IGF-dependent and -independent activities. Concerning an IGF-dependent mode of action, the attenuated platelet activation by the MACC1 positive SW620 cells was an unexpected finding that finally helped us to identify IGFBP2 as the responsible functional protein in the cell supernatant. Notably, an interplay of IGFBP2 and platelet activation has not been reported yet. Our data refer to a scavenging effect for IGF-I and, thus, slightly attenuated platelet activation by cells overexpressing IGFBP2. Platelet activation by tumor cells is a key issue to promote metastatic spread. Nevertheless, the attenuated platelet activation by IGFPB2 does not appear contradictory to the higher malignancy of MACC1-driven cells for several reasons. For instance, our data refer to a focused in vitro approach of tumor cell/platelet interaction accentuating certain differences in activation potential, e.g., indicated by the onset time of aggregation, while, in vivo, several factors additionally contribute to platelet activation. Basement fragments, such as subendothelial collagen, exposed to the blood flow in the process of tumor cell intravasation and tissue invasion potentially promote the process of platelet activation, but cannot be simulated in vitro. Furthermore, platelet activation is a vital part but is also covered by the procoagulant behavior of the cells. Activation of the plasmatic coagulation cascade was pronounced and independent of the IGFBP2 or MACC1 deregulation shown in the thrombin generation assay data.

The IGF-independent activities of IGFBP2 appear complex in an oncogenic network, affecting a series of signaling pathways for tumor growth and progression. At the functional level, the downregulation of IGFBP2 resulted in reduced migration of SW620 cells; similar findings have recently been shown using other colon cancer cells [30]. However, the molecular mechanistic background remains to be elucidated. Integrins appear as key mediators for the mechanotransduction of IGFBP2-related effects. Integrins have been described as cellular collectors for extracellular IGFBP2 to transmit the pro-tumorigenic signaling into the cell, e.g., by inhibiting the tumor suppressor activity of PTEN [36,37]. In these terms, IGFPB2 was reported to induce the nuclear translocation and activation of NF-κB through the PI3K/Akt pathway, inducing EMT and invasive character in pancreatic ductal adenocarcinoma [38]. IGFPB2 was revealed as a downstream target of overexpressed HSP27 in hepatocellular carcinoma cells, driving tumor proliferation, migration, and invasiveness by inducing vimentin, snail, and β-catenin [39]. The functional relationship of IGFBP2, NF-κB, and β-catenin fostering Wnt pathway has also been shown in CRC [31,40]. The cellular localization of IGFBP2 at the colonic crypts of normal mucosa and the higher expression levels in cancer tissues refer also to a role of IGFBP2 in stem cell formation [40]. Consequently, the spectrum of probable IGFBP2 activities is wide and it is a matter of speculation how IGFBP2 has affected the metastatic spread of SW620 cells. Thus, a detailed mechanistic answer requires further studies.

Driving the β-catenin/Wnt pathway and EMT is an emerging similarity shared by IGFPB2 and MACC1 in fostering tumor malignancy; however, both have not been functionally related yet. Although this functional relationship has been enlightened by our data, further studies are needed to decode this on a molecular and regulatory basis. However, the functional combination of IGFBP2 and MACC1 sheds a new light on previous studies, which emphasized either IGFBP2 or MACC1 in their individual roles as prognostic biomarkers in several solid tumor entities. It will be interesting to elucidate whether other cancer cell entities share IGFBP2 and MACC1 as pro-tumorigenic mediators for a common and synergistic consideration in future studies.

## 4. Materials and Methods

### 4.1. Cell Lines

Human colorectal cancer cell line SW620 (RRID:CVCL_0547) (American Type Culture Collection, Manassas, VA, USA) was grown in Dulbecco’s modified Eagle’s Medium (DMEM) (Sigma Aldrich, St. Louis, MO, USA) with 10% (*v*/*v*) FCS, 2% L-glutamine, and 1% sodium pyruvate. Cells were incubated at 37 °C in a humidified atmosphere containing 5% CO_2_. Cells were detached at 90% confluence using a solution of EDTA (0.2 g/L EDTA × 4 Na) for 10 min at 37 °C. All reagents were from Thermo Fisher Scientific Inc. (Waltham, MA, USA). The MACC1 knockout clones were generated by CRISPR/Cas9-mediated gene editing, as described earlier [3]. Gene transfer of luciferase and IGFBP2 shRNA was performed by lentiviral transduction. The plasmid PLKO-mcherry-luc-puro-Renilla was a gift from Carl Novina (Addgene plasmid # 29783; http://n2t.net/addgene:29783, accessed on 28 December 2015; RRID: Addgene_29783) [3]. The shRNA-coding shlenti constructs were purchased from Origene (Rockville, ML, USA). Successfully transduced clones were selected by FACS and maintained using 1 µg/mL puromycin. Cells were routinely tested for absence of mycoplasma by qPCR; all experiments were performed with mycoplasma-free cells. SW620 cells were authenticated using short tandem repeats.

### 4.2. Preparation of Washed Platelets

Platelet-rich-plasma was obtained from Institute for Experimental Hematology and Transfusion Medicine, University of Bonn, Medical Centre, in accordance to the declaration of Helsinki. Isolated human platelets in buffer were prepared from platelet-rich plasma by centrifugation (670× *g*, 10 min, 22 °C) and resuspension (400 × 10^6^ Plts/mL) in recalcified (1 mM) platelet buffer (10 mM HEPES, 137 mM NaCl, 2.6 mM KCl, 1 mM MgCl_2_, 13.8 mM NaHCO_3_, 0.36 mM NaH_2_PO_4_, 5.5 mM D-glucose), as described previously [41].

### 4.3. Light Transmission Aggregometry

Measurement of tumor-cell-induced platelet aggregation was performed by light transmission aggregometry using an APACT-4004 aggregometer (Haemochrom Diagnostica, Essen, Germany). Platelets were prepared as described in the previous section “Preparation of washed platelets”. Platelet aggregation was induced by 1 × 10^4^ tumor cells/mL at 37 °C in adequate cuvettes stirred continuously at 1000 rpm. Aggregate formation was measured by light transmission, with buffer set as 100% and platelets in buffer as 0% reference values. Aggregates induced by tumor cells are composed of platelets and tumor cells.

### 4.4. Platelet Dense Granule Secretion Assay

Tumor cells were detached with EDTA and resuspended in PBS. Platelets (400 × 10^6^ Plts/mL) were activated with 1 × 10^4^ tumor cells/mL for 20 min. Platelet ATP secretion from dense granules was assessed by luminescence measurement using a luciferin-based ATP-Determination Kit (Thermo Fisher Scientific, Waltham, MA, USA) and a FLUOstar Optima plate reader (BMG Labtech, Ortenberg, Germany). Platelets (400 × 10^6^/mL) were activated with thrombin receptor activator peptide 6 (TRAP-6, 42 µM) for 5 min, and ATP was subsequently quantified.

### 4.5. Platelet Tumor Cell Adhesion Assay

Platelets were labeled with calcein AM at a final concentration of 2 µM for 30 min at 37 °C and, afterwards, washed with platelet buffer. Confluent tumor cells in 96-well plates were washed with PBS and 400 × 10^6^ Plts/well were added. After shaking for 25 min, unbound platelets were removed by washing with PBS. Platelets attached to tumor cells were lysed with 100 µL Triton X-100 (1% in PBS) and transferred to black 96-well plates. Fluorescence was quantified using a plate reader (BMG POLARstar, BMG Labtech, Ortenberg, Germany).

### 4.6. Reverse Transcription and qRT-PCR

RNA isolation was performed using the Universal RNA extraction kit (Roboklon, Berlin, Germany). A quantity of 50 ng of total RNA was used for MuMLV-based cDNA synthesis (Biozym, Vienna, Austria). Primers for MACC1 were described elsewhere [1]. The primers for human IGFBP2 are forward: 5′-GCC CTC TGG AGC ACC TCT ACT-3′ and reverse: 5′-CAT CTT GCA CTG TTT GAG GTT GTA C-3′. All primers were synthesized at BioTeZ Berlin-Buch. qRT-PCR was performed as described earlier [3]. In brief, after initial denaturation at 95 °C, the amplification was performed for 40 cycles of denaturation (5 s; 95 °C) and a combined primer annealing and elongation step (45 s; 60 °C). Data were analyzed with LightCycler 480 Software release 1.5.0 SP3 (Roche Diagnostics, Penzberg, Germany). Mean values were calculated from duplicates. Each mean value of the expressed gene was normalized to its G6PDH level.

### 4.7. Western Blot

Tumor cells were centrifuged (450× *g*, 4 min, 22 °C) and lysed with cell extraction buffer (Thermo Fisher) supplemented with 0.1 mM PMSF and protease inhibitors (1 μg/mL aprotinin, 1 μg/mL leupeptin) (Life Technologies, Carlsbad, CA, USA). Precast gels with a polymerization degree of 10% were used (Mini-PROTEAN^®^ TGX™ Stain-Free™; Bio-Rad Laboratories GmbH, Munich, Germany). Proteins were transferred to Roti^®^-PVDF membrane (Carl Roth GmbH, Karlsruhe, Germany). The membrane was blocked with skimmed milk powder in Tris-buffered saline–Tween 20 (with 0.2% Tween 20) for 60 min, followed by three washing cycles of 10 min each. Afterwards, membranes were incubated with primary antibodies for IGFBP2 (Abcam, Berlin, Germany) for a total of 60 min at room temperature, and then incubated at 4 °C overnight. Membranes were rinsed again three times before applying the secondary anti-mouse IgG HRP-conjugated mAbs (Santa Cruz Biotechnology, Dallas, TX, USA) for 90 min. Primary antibody was diluted 1:5000 and secondary antibody was diluted 1:20,000. After rinsing the secondary antibodies, membranes were detected using Clarity™ECL Western Blotting Substrate (Bio-Rad Laboratories GmbH, Feldkirchen, Germany). For quantitative determination, either the StainFree™ technique was employed (Bio-Rad Laboratories GmbH) or normalization of proteins against the housekeeping protein α-tubulin. Pixel density analysis was performed with the IMAGE LAB software (Bio-Rad Laboratories GmbH).

### 4.8. Enzym-Linked Immunosorbent Assay

The human IGFBP2 and human IGF-I concentrations of the platelet releasates and tumor cell supernatant were determined with human enzyme-linked immunosorbent assays according to the manufacturer’s instructions (R&D Systems, Minneapolis, MN, USA).

### 4.9. Flow Cytometry

To determine tissue factor expression on SW620 cells, 5 µg anti-tissue factor goat polyclonal antibody (BioTechne, Wiesbaden-Nordenstadt, Germany) was added to 1 × 10^5^ tumor cells, respectively. Afterwards, secondary FITC-labeled anti-goat IgG was added to 1 × 10^5^ tumor cells. After washing with PBS twice, 1 × 10^4^ cells were measured by flow cytometry with a Guava easy Cyte 11 HT (Merck Millipore). Mean and standard deviation were calculated of the median fluorescence intensities of each experiment, determined with InCyte Software (Merck Millipore, Billerica, MA, USA).

### 4.10. Thrombin Generation Assay

Tumor-cell-induced thrombin generation was evaluated using a fluorogenic thrombin generation assay. Platelets were prepared and incubated with tumor cells, resulting in a final concentration of 1 × 10^4^ cells/mL. After adding the fluorogenic substrate Technothrombin THA SUB (Technoclone GmbH, Vienna, Austria) and recalcification (1 mM CaCl_2_), the kinetics of the subsequent thrombin-mediated Z-Gly-Gly-Arg-AMC cleavage were measured immediately and results were converted into thrombin concentrations using the evaluation software provided by Technoclone GmbH.

### 4.11. Cell Migration Assay

In total, 6 × 10^5^ cells were seeded on uncoated 24-well plates (Starlab GmbH, Hamburg, Germany). After 6 h, a scratch was conducted with a 10 µL pipette tip (Starlab GmbH), medium was removed, wells were washed once with PBS, and fresh FCS-free medium with platelets (4 × 10^6^/mL) or only platelet buffer was added. Wound healing was observed every 24 h for 72 h with 10-fold magnification using an Axiovert 200 microscope (Carl-Zeiss, Jena, Germany). Migration speed was quantified as reduced scratch wound over time.

### 4.12. Cell Invasion Assay

For cell invasion, Nunc™ Polycarbonat-Transwell-Inserts (Thermo Scientific GmbH, Langenselbold, Germany) were coated with 15 µg/cm^2^ collagen type I (Discovery Labware Inc, Bedford, TX, USA). The lower chamber was filled with 5% FCS medium as a chemoattractant. In total, 5 × 10^5^ cells were suspended in serum-free media with platelets (40 Mio/mL), with supernatant of platelets after activation with TRAP-6 (40 × 10^6^/mL), or with platelet buffer only and added to the filter in the insert. After incubation for 3 h, cells in both chambers were quantified by flow cytometry (Guava easy Cyte 3 HT). Additionally, cells were fixed on the filter, permeabilized, stained with Trypan blue, and quantified with a microscope (Zeiss Axiovert 200M) with 10-fold magnification.

### 4.13. Genome-Wide Expression Analysis

Differential gene expression was studied with whole transcriptome microarrays (Human Gene 2.0 ST array, Affymetrix, Santa Clara, CA, USA) to identify genes that are differentially regulated in SW620/sh control (MACC1 endogenously high) compared with SW620/shMACC1 cells. The total number of samples analyzed was six: three replicates for cells with high endogenous MACC1 expression after stable gene transfer of control shRNA and three replicates for cells that showed reduced MACC1 gene expression after stable gene transfer of a MACC1-specific shRNA (both shRNAs: OriGene Technologies, Rockville, MD, USA). For this, three different passages of each cell clone, SW620/sh control and SW620/shMACC1, were harvested and total RNA was isolated with the RNeasy Mini Kit (Qiagen, Düsseldorf, Germany; including DNA digestion). For microarray analyses, only RNA isolates with high quality, determined using the Agilent 2100 Bioanalyzer (Agilent Technologies, Santa Clara, CA, USA) with an RNA integrity number > 9.0, were used. Preparation of fragmented and labelled cDNA for analysis was performed using the GeneChip WT PLUS Reagent Kit (Affymetrix) following the manufacturer´s instructions. Hybridization, washing, and staining, finalized by scanning, were performed using Affymetrix GeneChip Hybridization Oven640, GeneChip Fluidics Station 450, and Affymetrix GeneChip Scanner 7G (all Affmetrix). Data were processed and analyzed using the Expression Console and Transcriptome Analysi Console (Affymetrix/Applied Biosystems). Statistically significant (*p* ≤ 0.05) differentially expressed genes were mined for candidates with influence in platelet activation or aggregation. The microarray data were submitted to the National Center for Biotechnology Information (NCBI) Gene Expression Omnibus database (geo@ncbi.nlm.nih.gov, https://www.ncbi.nlm.nih.gov/geo/, accessed on 2 June 2021). The data are available using GSE175995.

### 4.14. Immunohistochemistry

Immunohistochemistry was performed as described elsewhere [42]. Briefly, cryosections were stained with rabbit anti-human-specific cytokeratin-19 antibody (Acris Antibodies, Herford, Germany) and detected with DAB Substrate Kit (Vector Laboratories, Burlingame, CA, USA) after incubation with horseradish peroxidase-coupled anti-rabbit antibody (Promega, Madison, WI, USA).

### 4.15. Animal Experiments

All in vivo experimental work was performed in accordance with the United Kingdom Co-ordinating Committee on Cancer Research (UKCCCR) guidelines and approved by responsible local authorities (G0333/18, State Office of Health and Social Affairs, Berlin, Germany). Six 8-week-old female, nonobese diabetic (NOD), severe combined immunodeficiency (SCID) mice (n = 10 per group; Janvier Labs, Le Genest-Saint-Isle, France) were injected with 1 × 10^6^ SW620/shctrl or SW620/shIGFBP2 cells (EPO GmbH, Berlin, Germany) in 30 µL sterile PBS into the spleen. The experiment was terminated and mice were sacrificed by cervical dislocation when the ethical endpoint was reached by first animals, e.g., overall health status, tumor size, or weight loss. The livers (the metastasis target organ) were removed and shock frozen in liquid nitrogen. Cryosections were performed prior to isolation of genomic DNA (DNA/RNA/Protein extraction kit, Roboklon) for quantification of human satellite DNA [43] and for CK19 staining.

### 4.16. Statistical Analysis

Comparisons were performed using the software Prism™ version 9 (GraphPad Software, San Diego, CA, USA). Student’s *t*-test was used to compare two groups, and one-way analysis of variance (ANOVA) and Dunnett’s post hoc test were used for three or more groups. * *p* < 0.05; ** *p* < 0.01; *** *p* < 0.001; **** *p* < 0.0001 indicated statistical significance.

## 5. Conclusions

The unexpected finding that MACC1, a marker for increased malignancy, is inversely related to platelet activation potential of SW620 CRC cells finally helped to identify IGFBP2 as a functional downstream molecule with relevance for platelet communication. Consequently, our data provide evidence for a not yet recognized functional axis of MACC1 and IGFBP2, which also appears relevant in other CRC cell lines. Notably, deregulation of MACC1 or IGFBP2 did not affect tumor-cell-induced thrombin formation, which is considered of overriding importance for tumor-related thrombosis and metastasis. Thus, these data are not contradictory to the accepted role of platelets in malignancy and metastasis of tumor cells, but underscore the multifaceted molecular pathways of platelet activation in an oncological context.

## Data Availability

Data of the whole genome array of the SW620 MACC1 and SW620 MACC1 k.d. cells were submitted to the National Center for Biotechnology Information (NCBI) Gene Expression Omnibus database (geo@ncbi.nlm.nih.gov, https://www.ncbi.nlm.nih.gov/geo/, accessed on 2 June 2021), available using GSE175995. All other data of this study are available from the corresponding authors upon request.

## Supplementary Materials

The following are available online at https://www.mdpi.com/article/10.3390/ijms222212195/s1.
