# Supplementary material for: Insulin-like Growth Factor Binding Protein-2 (IGFBP2) Is a Key Molecule in the MACC1-Mediated Platelet Communication and Metastasis of Colorectal Cancer Cells
Source: Int J Mol Sci. 2021 Nov 11;22(22):12195. doi: 10.3390/ijms222212195 (PMC8624049; doi:10.3390/ijms222212195)
Supplement: Supplementary file 1 [file ijms-22-12195-s001.zip › ijms-1433498-supplementary.pdf]

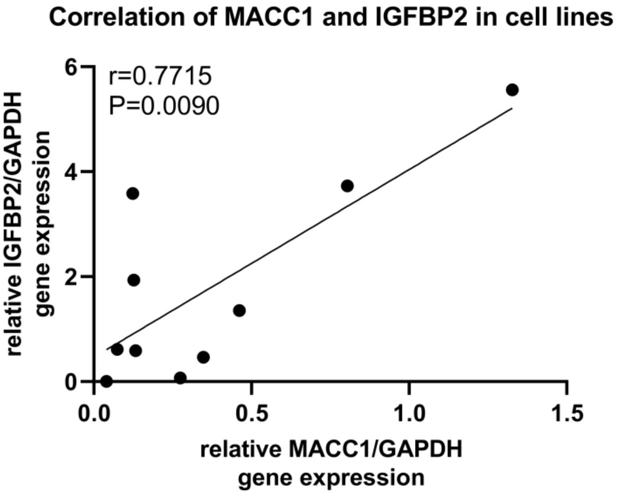

Supplementary Figure S1: Correlation of MACC1 and IGFBP2 expression levels in ten different CRC cell lines

Human colorectal cancer cell lines SW48, WiDr, DLD-1, SW403, HCT116, Colo205, HCT15 and LoVo cells were cultivated in Roswell Park Memorial Institute (RPMI)-1640, HCA-7 and LS174T cells were grown in Dulbecco's modified Eagle's Medium (DMEM), supplemented with 2 mM L-glutamine and 100 mg/L sodium pyruvate or 2mM L-glutamine and 1% NEAA, respectively. Cells were incubated at 37°C in a humidified atmosphere containing 5% CO_2.

MACC1 and IGFBP2 expression levels were determined in the same samples of a variety of several CRC cell lines by qRT-PCR, normalized using GAPDH and sorted by ascending MACC1/GAPDH mRNA levels. Expression values were analyzed using GraphPad Prism 9.

We investigated the correlation of MACC1/GAPDH vs IGFBP2/GAPDH and found a positive, statistically significant correlation with a Pearson r of 0.7715 and a P value of 0.009.
